# Retraction: G-Protein–Coupled Receptor 30 and Estrogen Receptor-α Are Involved in the Proliferative Effects Induced by Atrazine in Ovarian Cancer Cells

**DOI:** 10.1289/ehp.11297RET

**Published:** 2014-02-01

Retraction of: Environ Health Perspect 116:1648–1655; doi:10.1289/ehp.11297

The authors discovered that inadvertent errors occurred in sorting the files related to the presentation of the Western blots in Figures 6 and 8 and requested that their paper be retracted. The authors stand by the soundness of the study and have advised the Editor-in-Chief of their intention to submit an amended manuscript, which incorporates appropriate changes, for consideration for publication in a future issue of the journal. The authors apologize for any inconvenience caused.

